# Variation of the Nutritional Composition and Bioactive Potential in Edible Macroalga *Saccharina latissima* Cultivated from Atlantic Canada Subjected to Different Growth and Processing Conditions

**DOI:** 10.3390/foods12081736

**Published:** 2023-04-21

**Authors:** Bétina Lafeuille, Éric Tamigneaux, Karine Berger, Véronique Provencher, Lucie Beaulieu

**Affiliations:** 1 Département de Science des Aliments, Faculté des Sciences de l’Agriculture et de l’Alimentation (FSAA), Université Laval, Québec, QC G1V 0A6, Canada; betina.lafeuille.1@ulaval.ca; 2 Institut sur la Nutrition et les Aliments Fonctionnels (INAF), Québec, QC G1V 0A6, Canada; etamigneaux@cegepim.ca (É.T.); veronique.provencher@fsaa.ulaval.ca (V.P.); 3 Centre Nutrition, Santé et Société (NUTRISS), Université Laval, Québec, QC G1V 0A6, Canada; 4 École des Pêches et de L’aquaculture du Québec, Cégep de la Gaspésie et des Îles, Québec, QC G0C 1V0, Canada; 5 Merinov, Grande-Rivière, QC G0C 1V0, Canada; karine.berger@merinov.ca; 6 École de Nutrition, Faculté des Sciences de l’Agriculture et de l’Alimentation (FSAA), Université Laval, Québec, QC G1V 0A6, Canada; 7 Québec-Océan, Université Laval, Québec, QC G1V 0A6, Canada

**Keywords:** brown macroalgae, wild, season, blanching, steaming, dried, antioxidant activity

## Abstract

Macroalgae are a new food source in the Western world. The purpose of this study was to evaluate the impact of harvest months and food processing on cultivated *Saccharina latissima* (*S. latissima*) from Quebec. Seaweeds were harvested in May and June 2019 and processed by blanching, steaming, and drying with a frozen control condition. The chemical (lipids, proteins, ash, carbohydrates, fibers) and mineral (I, K, Na, Ca, Mg, Fe) compositions, the potential bioactive compounds (alginates, fucoidans, laminarans, carotenoids, polyphenols) and in vitro antioxidant potential were investigated. The results showed that May specimens were significantly the richest in proteins, ash, I, Fe, and carotenoids, while June macroalgae contained more carbohydrates. The antioxidant potential of water-soluble extracts (Oxygen Radical Absorbance Capacity [ORAC] analysis–625 µg/mL) showed the highest potential in June samples. Interactions between harvested months and processing were demonstrated. The drying process applied in May specimens appeared to preserve more *S. latissima* quality, whereas blanching and steaming resulted in a leaching of minerals. Losses of carotenoids and polyphenols were observed with heating treatments. Water-soluble extracts of dried May samples showed the highest antioxidant potential (ORAC analysis) compared to other methods. Thus, the drying process used to treat *S. latissima* harvested in May seems to be the best that should be selected.

## 1. Introduction

The most recent concept of “sustainable food” no longer considers only environmental factors but must integrate economic and social factors. The aim is to enable the production of food for the growing world population while providing food security and preserving the environment [1,2]. Thus, new foods that meet these requirements are coming under the spotlight, as is the case for macroalgae.

Traditionally consumed in Asia, macroalgae still hold an important place in Asian culture, food, and economy. In 2019, China and Indonesia were the world leading macroalgae producers and consumers with 57% and 28%, respectively, of world seaweed biomass production (farmed and wild), with 99% of these volumes produced by sea farms [3]. In the Western world, macroalgae remain largely underexploited, except for a few specific coastal regions such as Iceland, Ireland, France, Denmark, Norway, the United States, and Canada [4,5]. Based on their pigmentation, macroalgae are divided into three groups: *Ochrophyta, Phaeophycea* (brown), *Chlorophyta* (green), and *Rhodophyta* (red) [6]. Between 9000 and 15,000 species are known and recorded [6,7]. Among them, the brown *Saccharina latissima* (*S. latissima*), also called *Saccharina longricruris* [8] or *Laminaria saccharina* [9], or royal kombu (vernacular name), is one of the most common seaweeds in Asian and North Atlantic coasts [10]. This species lives in the intertidal zone from 1 m to 15 m below the water surface in areas where they can attach to the rocky seabed [11]. Brown macroalgae, such as those of genuses *Laminaria* and *Saccharina* have a healthy nutritional composition, with low-fat (0.3–2.9%), a high polysaccharide content (38–61%), including fiber, minerals (15–45%) and a variable amount of proteins (3–21%) [5]. Potential bioactive compounds found in brown seaweeds include peptides, sulfated polysaccharides (alginates, fucoidans, laminarans), or secondary metabolites (carotenoids and polyphenols). These compounds exhibit various bioactive activities including antioxidant, antibacterial or hypotensive effects [7,10,12,13,14,15,16].

However, the chemical and bioactive composition of seaweeds is highly variable, depending on species and environmental factors (geographical location, season, water temperature, salinity, and light intensity) [17,18,19,20]. Indeed, the life of macroalgae from temperate areas is characterized by a strong seasonality in their growth and reproduction patterns. For some Atlantic macroalgae, fast growth is observed in winter and spring, whereas in summer and fall, when reproduction occurs, the growth rate is much lower [17,21,22,23]. Previous studies of *S. latissima* have shown that, in the North Sea and Norway, the predominant growth period is in late winter and early spring, while the growth rate decreases substantially but does not stop in July [17]. Trials conducted in the Gulf of Saint-Lawrence (QC, Canada) in 2012, confirmed that when *S. latissima* is cultivated on subsurface longlines, maximum frond size is observed in June and progressively decreases beginning in July, reaching a minimum in September [23]. During late winter and spring, the rapid growth is the result of active photosynthesis enabled by the growing photoperiod and light intensity, and by high concentrations of nutrients in seawater [17,23,24,25]. Indeed, inorganic carbon is the major substrate of photosynthesis in marine macroalgae, while nitrogen uptake enables the synthesis of proteins (such as RuBisCo) involved in this biological process, and minerals have multiple roles, such as the synthesis of ferredoxin (Fe associated with protein) or chlorophyll (which contains Mg) [17,24,25]. During the summer, the growth rate of *S. latissima* declines due to a combination of phytoplankton growth and water stratification that strongly reduces the availability of nutrients for cultivated specimens [26]. In addition, from late June/early July, planktonic larvae of bryozoans (such as *Membranipora membranacea*) attach to blades and grow colonies that limit light absorption by macroalgae [17,23].

Previous work on wild *S. latissima* harvested in Quebec in May and August 2005 showed significant seasonal differences in the composition of the tissues, with higher protein and mineral contents in May than in August [27]. In another study performed on *S. latissima* harvested from six different locations in Quebec between June and August 2015, the month and site of the harvest were also identified as factors determining the nutritional quality of the fronds [28]. Indeed, higher protein and lipid contents in July compared to June were reported for the same location (Îles-de-la-Madeleine, QC, Canada). Bioactive compounds and bioactivities are also affected by factors such as time of the year and environment. Previous work carried out on *S. latissima* aqueous extracts harvested in the Newport area (Chandler, QC, Canada) between October and December 2015 detected variability in Oxygen Radical Absorbance Capacity (ORAC) values of 27.52 TE mmol g^−1^ [13]. A study performed in Quebec on *Saccharina longricruris* (*S. latissima*) determined that bioactive aqueous extracts contained peptides from various proteins such as ubiquitin or histone [29]. With the varying nutrient availability in seawater inducing variations in protein synthesis, the seasons might influence the bioactive potential of *S. latissima*.

Two factors make macroalgae attractive for processing: (1) seaweeds have a high moisture content (>75%) and thus are quite perishable when sold fresh [10,30], and (2) macroalgae are more likely to be consumed as processed food [3]. Drying is one of the earliest and most common methods used for food preservation [10,30,31,32]. In order to stabilize freshly harvested seaweed for long-term storage, different drying methods such as sun-drying, oven-drying (hot-air-drying), or freeze-drying have been investigated [10,30,32]. Of these, oven-drying does not require hot, sunny weather, it allows better control of drying parameters compared to sun-drying, and it is less expensive than freeze-drying [10,32]. Heat-aided drying results in faster drying but could negatively affect food texture, color, nutritional value, or bioactive potential [10,33]. Indeed, carotenoids or polyphenols are known to be highly sensitive to heat [10,34] and a previous study showed a total phenolic content reduction in three brown seaweeds even when a low temperature was used (20 °C) [35]. On the other hand, the nutritional composition of *S. latissima* is no more affected when dried at high temperature (70 °C vs. 25 °C or 40 °C) [30]. Freezing is also a very common preservation method to increase the shelf life of foods by slowing or stopping microorganism metabolism and enzyme activity [31,32]. When the storage temperature is below the freezing point, ice crystals form within the food causing concentration of nutrients and cell wall damage. The latter increases nutrient bioavailability, denaturation of proteins, color and texture changes [31,32,36]. A study conducted on *Alaria esculenta* revealed losses in amino acid content in the tissues when specimens were frozen (−20 °C) and thawed (5 °C) [37]. 

Even if drying and freezing methods are largely used alone and are effective, blanching prior to drying or freezing could improve these methods and limit food deterioration [31,32,38]. Blanching is one of the most common treatments applied to fruits and vegetables to sanitize them by destroying microorganisms or insects and inactivating certain enzymes such as lipoxygenases [36,38,39]. Briefly, two blanching methods are wildly used: immersing food in boiling water (called blanching in this study) or exposing food to steam (steaming) [33,39,40,41]. The main issues with high heat treatments such as blanching and steaming are the loss of heat sensitive molecules such as polyphenols and carotenoids, or the loss of soluble molecules by leaching [39,42]. In *S. latissima*, mineral loss reached values > 70% when fronds were blanched [41]. Steaming is known to reduce this phenomenon as observed by Nobosse and colleagues [43] with the terrestrial plant *Moringa oleifera* L., where mineral leaching with steaming was only 11%. However, it was shown that carotenoids are negatively affected when *Himanthlia elongata* are steamed [44]. 

In addition to changes that may be induced by processing, the seaweed growth environment also appears to influence macroalgae modifications [10]. Despite the existence of several studies on processing effects on *S. latissima*, the simultaneous impact of growth conditions and common processing methods on nutrition, minerals, potential bioactive compounds and bioactive activity has not been previously reported. From the point of view of algal farmers and processors, the effects of harvest time and processing methods on the nutritional and bioactive compounds of the macroalgae are important issues. The objectives of the present study were: (1) to investigate the impact of the harvest month (May and June) on the chemical (lipids, proteins, ash, carbohydrates, fibers), mineral (I, K, Na, Ca, Mg, Fe) and bioactive compound composition (alginate, laminarans, fucoidans, carotenoids, polyphenols), and in vitro antioxidant potential of the macroalgae *Saccharina latissima*; and (2) evaluate the effect of different food treatments (blanching, steaming, drying) on chemical, mineral and bioactive composition, and in vitro antioxidant potential. 

## 2. Materials and Methods

### 2.1. Macroalgal Biomass

**Figure 1:**
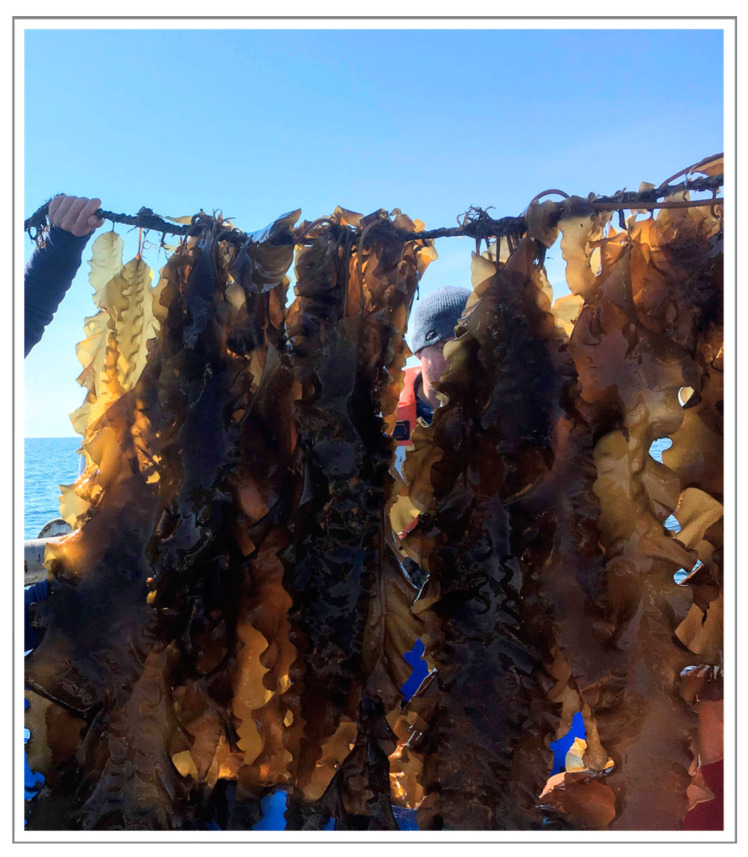
Harvesting of *Saccharina latissima* (*S. latissima*) cultivated on subsurface aquaculture longlines on 29 May 2019, at a marine farm in Paspébiac, Gaspé. Photographs: AGHAMW, Paspébiac Bay, QC, Canada.

The macroalgae *Saccharina latissima* (Figure 1) was cultivated on the AGHAMW (Mi’kmaq and Maliseet Halieutics Management Association) marine farm in Paspébiac Bay (Baie-des-Chaleurs, QC, Canada). Briefly, nylon twines were seeded with macroalgae spores and the plantlets were grown for 5 weeks in a marine hatchery. During fall 2018, when juvenile kelp reached 4 mm long, the twines were transferred to the marine farm, attached to the aquaculture longlines and kept at 4 m depth. In November, the culture lines were lowered and maintained at a 7 m depth until 10 and 16 May 2019, when they were raised to 4 m below the surface. *S. latissima* blades were collected twice, on 29 May and on 19 June 2019.

### 2.2. Processing of the Blades

Before processing, macroalgae fronds were cleaned with fresh water. Thereafter, the fronds were trimmed to remove the stipe, epibionts, and damaged parts of the blades. Only the clean proximal part of the blades was kept for analysis (the first 100 cm above the stipe). All blade samples were stored at 4 °C overnight and processed the next day. Three different treatments were applied for *S. latissima*: boiling, steam bleaching, and air drying. Fresh freezing seaweeds were used as controls.

Based on currently used methods in the Quebec algal industry, boiled batches were created by boiling the seaweed in salt water (30 g/L, ≃100 °C) for 1.5 min, and steam-bleached batches were generated by steaming the fronds in a Vulcan^®^ steamer (Vulcan^®^, Baltimore, MD, USA) for 1.5 min. Immediately after those treatments, macroalgae were cooled in iced fresh water for 2 min and then drained manually. Air-dried batches were dehydrated at 40 °C in a dryer (Hamilton Beach, Glen Allen, VA USA) for about 5 h. Finally, and after each treatment, all samples were ground into 1 to 0.5 cm^2^ flakes. For control batches, the grinding step was performed before freezing at −80 °C. All samples were stored at −80 °C until further use. 

### 2.3. Nutritional Composition (Lipids, Ash, Proteins, Carbohydrates, Fibers) and Mineral Composition

Prior to nutritional characterization, frozen macroalgae were lyophilized and ground into a fine powder (BFP660, Breville, Sydney, Australia). Then, the moisture was determined by using method no. 950.46 AOAC (Association of Official Analytical Chemists) 2008 and ash content was obtained using method no. 938.08 AOAC 2008. The quantification of calcium, iron, magnesium, potassium, and sodium was determined by adding chloric acid in ash and identified by flame atomic absorption spectroscopy (220FS Varian, Palo Alto, CA, USA), according to AOAC 2008 method no. 968.08. For iodine determination, samples were dissolved in hot milli-Q water (90 °C) and iodine content was quantified using iodine ion selective electrodes (Thermo Scientific 258508, Waltham, MA, USA). The Kjeldahl method was used to obtain the amount of crude protein in the samples (AOAC 2008, no. 988.05). The protein factor was set at 5.0 according to Angell et al. [45]. Lipid measurement was performed following the Bligh and Dyer method [46]. The total estimate of carbohydrate content was determined by difference according to the method of Wang et al. [47] and the fiber content was measured with a Megazyme kit (Bray, Ireland) according to AOAC 2008 method no. 985.29.

### 2.4. Potential Bioactive Compound Determination

Before bioactive compound determination, frozen macroalgae were lyophilized and ground. All measures were performed in triplicate.

#### 2.4.1. Total Phenolic Content 

The total phenolic content was obtained by colorimetry, following an adapted Folin-Ciocalteu method [48,49]. First, phenolic compounds were extracted from 0.5 g of macroalgae by accelerated solvent extraction with 95 °C water at 1500 PSI using the accelerated extraction system ASE-200, (Dionex, Camberley, Surrey, UK). The aqueous macroalgae extracts were mixed with 1.25 mL of 20% (*v/v*) aqueous Na2CO3 solution, 0.5 mL of Folin-Ciocalteu reagent, and 6 mL of deionized water. The samples were then incubated in the dark for 30 min at room temperature. Finally, the absorbance at 750 nm was read using a spectrophotometer (Spectrafluor Plus, Tecan, Thermo Scientific, Ottawa, ON, Canada). Results were expressed as milligram Gallic acid equivalent (GAE)/g dry extract [50].

#### 2.4.2. Carotenoids

The carotenoid content was measured by colorimetry. First, pigments were extracted from samples using absolute ethanol, containing 1.0% (*w/v*) of BHT (to avoid oxidation). Extraction, using 0.5 g of macroalgae samples, was performed with an accelerated extraction system ASE-200 (Dionex, Camberley, Surrey, UK) [51]. Then, ethanol was evaporated with a rotary evaporator (RV10, IKA, Wilmington, NC, USA) and extracts were dissolved again in 10 mL of petroleum ester. Finally, the absorbance of the extracts at 468 nm was read using a spectrophotometer (Genesys 20, Thermofisher, Waltham, MA, USA). The carotenoid concentration was obtained using the mass extinction coefficient of astaxanthin which is 233.5 L g^−1^ cm^−1^.

#### 2.4.3. Alginates, Fucoidans, and Laminarans

Alginate, fucoidan, and laminaran contents were determined in *S. latissima* samples as these polysaccharides are specific to brown macroalgae [27,52].

The content of both fucoidans and laminarans in macroalgae was obtained gravimetrically, following the protocol of Fletcher et al. [52]. Briefly, these polysaccharides were extracted by mixing 0.5 g of crushed and lyophilized macroalgae into Milli-Q water (for laminarans) or 0.1 M HCl (for fucoidans) at a ratio of 1:25 (*w/v*). The mixture was heated at 80 °C for 4 h, then cooled, centrifuged, and decanted. A series of 80% ethanol precipitations, decantations, and centrifugations produced the aqueous laminaran and fucoidan extracts. Quantification of these polysaccharides was then done using a preconditioned filter paper (oven heated to remove moisture and pre-weighed) and the weight was measured. 

The alginate content was also obtained by gravimetry, but the extraction was performed using the procedure of Honya et al. [53]. First, samples (0.5 g) were demineralized by acid hydrolysis, using 20 volumes of 0.5% (*v/v*) sulfuric acid solution for 2 h. Alginates were solubilized in a basic medium and extracted overnight at 40 °C with stirring. Then, alginates were precipitated with 10 mL of 1% calcium (*w/v*) to produce calcium alginate and transformed into alginate by adding 0.1 M HCl (1:25 *w/v*). The purification was carried out with successive washes with Milli-Q water, ethanol, and acetone. Finally, the quantification was conducted as described previously for laminarans and fucoidans. 

### 2.5. In Vitro Bioactive Potential

#### 2.5.1. Water-Soluble *S. latissima* Extracts

The water-soluble macroalgae extracts were obtained according to previously described methods [13,54,55]. Briefly, samples were mixed with phosphate buffer (20 mM, pH 7) at a ratio of 1:1.665 (*w/v*) for non-dehydrated and 1:16.667 (*w/v*) for dehydrated specimens, and agitated for 24 h at room temperature. Samples were then centrifuged at 4000× *g*, 4 °C for 45 min (Avanti^®^ J-E high-speed centrifuge, Beckman Coulter, Brea, CA, USA) and vacuum filtered. The supernatants were stored at 4 °C and extraction was performed again on the resulting pellets. Supernatants were pooled and brought to 80% saturated ammonium sulfate and stirred at 9 °C overnight to precipitate the proteins. Samples were centrifuged at 10,000× *g*, 4 °C for 60 min, pellets were solubilized in milli-Q water and dialyzed using a 1 kDa cut-off membrane unit (MWCO 1 kDa, Pur-A-Lyzer, Sigma-Aldrich, Saint-Louis, MO, USA) at 9 °C for 48 h. The effectiveness of the dialysis was controlled by conductivity measurements of the solution before and after. The resulting water-soluble macroalgae extracts were lyophilized and stored in the dark in vacuum bags at −20 °C until further analysis. Total protein content was quantified according to the Dumas method [56], using a TruSpec N nitrogen analyzer (Leco Corporation, St. Joseph, MI, USA) with a protein factor of 5.0 [45].

#### 2.5.2. Oxygen Radical Absorbance Capacity (ORAC) Assay

The ORAC analysis is a fluorescence-based method that measures the potential peroxyl radical scavenging capacity of macroalgae extracts, thus inhibiting fluorescein degradation and preventing fluorescence decrease. This assay was performed as described by Cao [57] and previously described methods [13,55]. First, water-soluble macroalgae extracts were dissolved in a phosphate buffer (75 mM, pH 7.4) at eight different concentrations (serial dilution from 5000 μg/mL to 39 μg/mL with a dilution factor of 2). Then, 25 μL of samples or Trolox standards (100 μM, 50 μM, 25 μM, and 12.5 μM) or 75 μL of blank (phosphate buffer) were added to black microplates (96 Well, Black U-Shape, Greiner Bio-One GmbH, Frickenhausen, Germany) and 150 μL of fluorescein (0.1 μM) was added to each well. The microplate was incubated in the dark at 37 °C for 30 min. The reaction was subsequently initiated by adding 50 μL of 2,2′-azobis (2-methylpropionamidine) dihydrochloride (AAPH) solution (150 nM) to each well. The fluorescence was read at a wavelength of 485 nm for excitation and 583 nm for emission at 37 °C and recorded every minute for 90 min using Synergy H1 (Biotek, Winooski, VT, USA). The calculated ORAC values were expressed in μmol equivalent Trolox per gram of the sample (μmol TE g^−1^).

#### 2.5.3. Ferric Ion Reducing Antioxidant Power (FRAP) Assay

The FRAP assay is another antioxidant test that measures the capacity of seaweed extracts to reduce a ferric oxide ion (Fe^3+^), by electron transfer, into a ferrous ion (Fe^2+^) which induces an intense blue color. It was performed according to the method of Kelman et al. [58] and adapted from previously described methods [12,55]. First, macroalgae extracts (<1 kDa) were solubilized in Milli-Q water at five different concentrations (500, 250, 100, 50, and 10 μg/mL). Then, a 180 μL volume of freshly prepared FRAP reagent was added to the wells of a 96-well microplate (Greiner Bio-One, Frickenhausen, Germany) and placed in the incubator at 37 °C for 10 min. Test concentrations or Trolox standards (50, 40, 25, 10, 5 μg/mL) or milli-Q water (blank) were added to the wells, and the microplate was incubated at 37 °C for 30 min. The absorbance was read at 593 nm using a Microplate Absorbance Spectrophotometer (xMark, Biorad, Hercules, CA, USA). The calculated FRAP values were expressed as μmol equivalent Trolox per gram of the sample (μmol TE g^−1^).

### 2.6. Statistical Analysis

All analyses (chemical, mineral, bioactive) were performed in triplicate. All values are expressed as the mean ± standard derivation (SD). Statistical analyses were mostly performed using SAS^®^ OnDemand for Academics (Cary, NC, USA). After using Fisher and Shapiro-Wilk tests to check for normality and homoscedasticity of samples, and when conditions were met, ANOVA tests were carried out to reveal significant differences between samples with a significance level set at *p* < 5%. These tests were followed by a Tukey test to discriminate the differences. For some cases where normality and homoscedasticity were not validated, Prism GraphPad^®^ software version 9.4.0 (San Diego, CA, USA) was used and a Kruskal-Wallis test followed by a multiple comparison Dunn test was conducted, also with a significance level set at 5%.

## 3. Results and Discussion

### 3.1. Chemical and Mineral Compositions

#### 3.1.1. Variations through Growth Conditions of Crude *S. latissima*

The chemical and mineral compositions of crude blades of *S. latissima* (*Sl*) used as control (kept frozen) are presented in Table 1 and Table 2, respectively. Some significant differences have been noted. For instance, the crude macroalgae *S. latissima* harvested in May were richer in proteins, minerals, iodine, and iron compared to those harvested in June, while the latter was richer in carbohydrates. No significant difference was observed in the lipid contents of blades harvested in May (1.88%) and June (1.63%). The protein content was significantly higher in samples from May, with concentrations varying from 10.86 to 11.14% (DW), while the content for June samples was significantly lower at 9.00%. At 43.06%, the average ash content was significantly higher in May samples compared to June samples (41.56%). Unlike other compounds, carbohydrate concentrations were significantly higher in June, with concentrations varying from 47.82% to 48.34%, whereas the average concentration for May samples was 44.05%. The fiber content was not significantly different between the two harvest months. Minerals analysis (Table 2) revealed significant differences for iodine (I) and iron (Fe). Both were higher in the blades harvested in May, 0.20 g I/100 g and 41.70 mg Fe/100 g, compared to 0.17 g I/100 g and 12.33 mg FE/100 g in June. No significant differences were observed for potassium (K), sodium (Na), calcium (Ca), and magnesium (Mg) contents.

These results are in accordance with the literature [5]. As presented in the introduction, the growth performance of macroalgae depends on environmental conditions such as the growth area, light, temperature, and the seasonal interactions of all factors [14,17,18], and this performance is particularly sensitive to substrate availability as nitrogen, inorganic carbon and minerals [17,24,25]. Thus, the abundance of protein and mineral content in May could be explained by the high concentrations of nutrients in seawater during winter and spring [10,17,21]. This difference, in favor of May, has already been observed in a previous study conducted on *S. latissima* cultivated in Norway which reported 11% to 26% higher protein content in May than in June [59]. Similarly, studies conducted on the same species cultivated in Denmark and Norway revealed a higher nitrogen content in the blade tissues in May than in June [60,61]. Furthermore, in early summer, the phytoplankton bloom combined with the stratification of the water column is known to progressively reduce the availability of nutrients in the surface layer [17,26], which favors the entry of macroalgae into a period of energy storage in June in preparation for both reproduction in the fall and the low light winter period [17,23,25]. This may result in slight increases in carbohydrate contents as observed in June in the present study. Handa et al. [60], working on *S. latissima* cultivated in Denmark and Norway, detected a higher carbon content in the blade in June (48%) than in May (35%) and similar observations were made by Nielsen et al. [61]. The results for lipids were similar to those obtained by Reissiger [59] who reported a higher average lipid content in May. 

For minerals, the Fe content in June was similar to that obtained for *S. latissima* cultivated in the North Sea in August (12.00 ± 0.13 g/100 g) [62]. The higher concentration in May seems related to higher seawater availability and photosynthetic rates [17,25,26]. Finally, the iodine (I) content was close to those previously reported in cultivated *S. latissima* from North Atlantic [62,63]. Iodine is a potent antioxidant in photosynthetic marine organisms. Thus, the high rate of photosynthesis may require good management of reactive oxygen species, explaining the higher amount in May compared to June [64].

**Figure 2:**
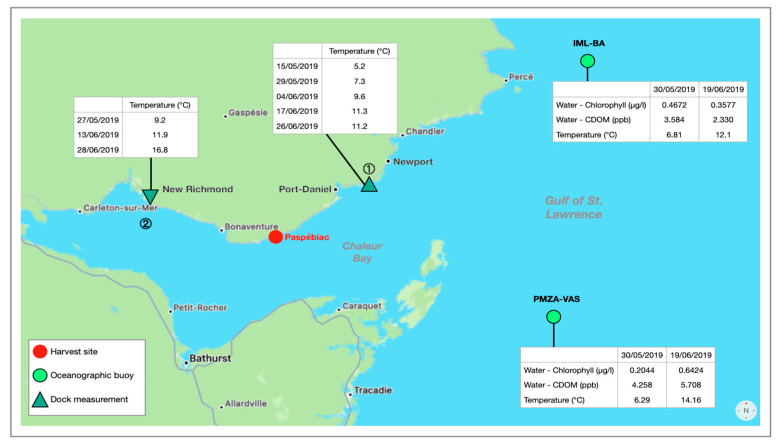
Map of the Baie-des-Chaleurs: location of the *S. latissima* harvesting site, oceanographic buoys and dock measurement areas, and data collected during May and June 2019. Credit data: https://ogsl.ca/conditions/?lg=fr, accessed on 6 April 2023. Credit map: Maps (Version 3.0) Apple Inc. (Cupertina, CA, USA).

These phenomena can also be explained based on data from two oceanographic buoys located in the Gulf of Saint-Lawrence (Figure 2), at the entrance of the Baie-des-Chaleurs (Quebec, Canada), not far from the Paspébiac harvest site (48°1.60′ N, 65°15’ W [65]). The northernmost IML-BA (48°35′ N, 63°53′ W) and the southernmost PMZA-VAS (47°46.998′ N, 64°2′ W) made various seawater measurements on 30 May 2019, one day after *S. latissima* was harvested, and on 19 June 2019, the day of the harvest [66]. For the PMZA-VAS buoy, chlorophyll in seawater was about 0.2044 µg/L in May versus 0.624 µg/L in June, and CDOM (Chromophoric Dissolved Organic Matter) was 4.258 ppb in May versus 5.708 ppb in June. Both chlorophyll quantification and CDOM measurement are related to phytoplankton abundance [67,68]. Thus, in the area of this oceanographic buoy, the chlorophyll concentration and CDOM were distinctly higher in June than in May, which could be correlated to a phytoplankton bloom. For IML-BA, the bloom did not seem to have occurred. Indeed, in May and June, the quantity of chlorophyll was 0.4672 µg/L and 0.3577 µg/L, while the CDOM was about 3.584 in May and 2.330 in June. This difference between the two oceanographic buoys could be due to their respective temperatures [69]. The reported temperature for PMZA-VAS in June was 14.16 °C but only 12.10 °C for IML-BA. Thus, environmental conditions near the macroalgae growing area would appear to be closer to those recorded by PMZA-VAS than by IML-BA. During May and June 2019, other temperature measurements were taken, but this time in dock areas in the Baie-des-Chaleurs. The first series of temperature measurements were taken between Newport, QC, Canada and Port-Daniel, QC, Canada (48°11.25′ N, 64°51.40′ W) (point ① Figure 2) and a second in New Richmond, QC, Canada (48°8.3′ N, 65°50.8′ W) (point ② Figure 2). In late May at point ①, close to the entrance of the bay, the temperature reached 7.3 °C whereas at point ② (New Richmond) it was 9.2 °C. Both are higher than those measured by oceanographic buoys in the same period. In mid-June, temperatures at points ① (13 June 2019) and ② (17 June 2019) were near 11 °C. However, in late June, the highest temperature was recorded at point ② (16.8 °C), while point ① was 11.2 °C. The bottom of the bay seems warmer. It is possible that the harvesting site benefited from beneficial marine currents with higher temperatures, which are more favorable for phytoplankton blooming, thus reducing marine nutrients in June.

#### 3.1.2. Variation through Food Processing of *S. latissima*

The DW chemical and mineral composition of processed *S. latissima* (blanched, steamed, dried, and frozen) is presented in Table 1 and Table 2, respectively. Statistical tests showed the presence of interactions between harvest months and treatments (*p*-value < 0.05).

- Blanching

Blanching treatment is known to reduce the total solids of vegetables and seaweeds through leakage from the biomass into the blanching liquid. Thus, water-soluble chemical components (such as carbohydrates, sugars, proteins, minerals, vitamins, etc.) could be lost during this process [38]. As shown in Table 1, and compared to crude seaweeds, blanched *S. latissima* contained the lowest mineral (ash) content for both harvest months (21.67 ± 0.01% DW in May and 27.53 ± 0.03% DW in June). These results agree with those of Akomea-Frempong, Perry et al. [39] for whole *S. latissima* blades where the ash content decreased with treatment of blanching at 100 °C for 30 s and the study of Nielsen et al. [41] where mineral content was measured at 44.51 ± 0.86% in fresh *S. latissima* thallus compared to 11.2 ± 1.4% in blanched thallus (80 °C, 120 s). The month of harvest also significantly impacted the ash content of blanched seaweed (higher in June than in May). For proteins and carbohydrates, the measured amounts significantly increased compared to those obtained in crudes of *S. latissima*. Thus, the protein content of blanched macroalgae ranged from 12.69 to 15.67% DW, and the carbohydrate content was about 60% DW. The increase in carbohydrates with blanching has already been observed in the literature, however, for protein content no significant difference was observed, but the trend was similar to our results [41]. Compared to blanched salted spinach, which contains about 2.4 g/100 g of fiber (wet basis), blanched *S. latissima* of this study provided approximately twice as much fiber [70]. Unlike minerals, protein, and carbohydrate contents are significantly higher in May compared to June. Even if the percentages of some chemical components in blanched seaweed are higher compared to crudes, it is interesting that this does not reflect the potential loss of biomass. Indeed, Nielsen et al. [41] highlighted the fact that between the fresh *S. latissima* and the blanched seaweeds (80 °C for 120 s) the total loss of biomass represented 53%. It is reasonable to think that this phenomenon similarly affects *S. latissima* in this study. 

For mineral composition (Table 2), I is significantly higher in June specimens compared to May but Mg and Fe are significantly higher in May. Compared to crude macroalgae, blanched specimens presented a significant loss of I, Mg (for both harvest months), K, and Fe (in May), no difference for Ca, and a significantly higher content of Na. The loss of minerals seems clearly linked to the leaching that could occur with this treatment [38,39]. For I, similar results were obtained in previous studies with losses of up to 83% of I content between fresh and blanched *S. latissima* [41,62]. 

Some brown macroalgae such as laminaranes are known to contain a high level of I [14,71]. Recommended intakes are about 1.1 mg per day, and an excess of that mineral could have adverse consequences on human health [14,71]. Reducing I in macroalgae through bleaching could therefore be a good strategy to allow individuals to consume more. The Ca content did not seem to be affected by the treatment which was also observed by Akomea-Frempong, Perry et al. [39]. However, no significant difference was observed in Mg content between samples. Akomea-Frempong, Perry, et al. [39] detected a loss of K with the blanching process, which is similar to our results in May. In June, although the difference between fresh and blanched macroalgae was not significant, the trend seems to show a decrease in K content, in line with previous work. The same authors previously reported that blanching produced a leaching of Na [39]. It should be noted that in this study, *S. latissima* was blanched in salted water (30 g/L), thus increasing the Na content of macroalgae.

- Steaming

Steaming of *S. latissima* (Table 1) was found to induce similar chemical composition results to blanching, with significantly higher amounts of proteins (14.66 ± 0.25% DW in May and 13.13 ± 0.11% DW in June) and carbohydrates (62.06 ± 0.27% DW in May and 63.34 ± 0.09% DW in June), and lower ash contents. The decrease in ash content was not different between blanching and steaming in May, but in June it was significantly lower for the steaming treatment (22.24 ± 0.17% DW). This phenomenon indicates that the leaching occurring in those two treatments was important and dependent on seaweed harvest time. Steaming, which is an alternative thermal method of blanching and can help reduce leaching [42], does not seem to limit mineral loss in these seaweeds. It is reasonable to think that the increase in protein and carbohydrate percentages was related to the loss of minerals. To the authors’ knowledge, no study has been conducted on macronutrient characterization of steamed *S. latissima* nor on fresh/frozen versus steamed macroalgae. However, the impact of steaming on our seaweeds could be compared to the results obtained on edible leaves of *Moringa oleifera* L., a terrestrial plant that is used to help fight malnutrition in developing countries [43]. In that study, the total amount of proteins, carbohydrates, lipids, and fibers was not significantly affected, but minerals were lost during the steaming process. However, ash leaching represented only 11% [43], whereas in seaweed it reached about 50%. In another study conducted on *Camellia sinensis* tea leaves, the authors reported changes in ash composition for which a 38% loss during steaming was observed [72]. Compared to steamed cauliflower, which contains, on a wet basis, about 2.56 g/100 g of protein and 0.71 g/100 g of ash, *S. latissima* provided less protein but twice as much mineral [73]). 

Compared to crude macroalgae, the mineral composition (Table 2) of steamed specimens contained significantly lower amounts of I (0.06 g/100 g in May and 0.07 in June) and Na (1.03 ± 0.12 g/100 g in May and 0.97 ± 0.06 in June), Mg in June (505.00 ± 27.00 mg/100 g) and Fe in May (31.67 ± 1.53 mg/100 g). The content of K and Ca was unchanged for both months. Changes in mineral composition depend on the harvest month and seem to be related to the total ash loss observed in the macronutrient composition. This phenomenon was observed in *Camellia sinensis* tea leaves where steaming induced a loss of Ca, Na, K, Mg, and Fe content [72]. It is interesting to note that in view of the percentages, the different minerals were not leached as much as during blanching. Steaming, therefore, seems to be better than blanching for retaining the minerals investigated [42].

- Drying

The overall chemical portrait of dried *S. latissima* was not different from crudes specimens (Table 1). Indeed, similar proportions of lipids (1.78% DW on average), proteins (10.38 ± 0.39% DW in May and 9.42 ± 0.11% DW in June), carbohydrates (44.86 ± 0.44% DW in May and 48.51 ± 0.42% DW in June), fiber (35.36% DW on average) for both months, and ash (in May specimens, 42.96 ± 0.15% DW) were found. Only minerals in June samples were slightly, but significantly, lower (40.09 ± 0.11% DW). Thus, in dried samples, similar differences between May and June were observed (higher in protein and ash in May and higher carbohydrate content in June). Compared to other heat treatments (blanching and steaming), the drying process resulted in better mineral preservation but lower percentages of proteins and carbohydrates, which might be a consequence of a higher ash rate. Seaweeds are known to be very sensitive to growth environments and treatments [5,14], and the available literature reflects that, with multiple studies investigating the chemical composition of dried *S. latissima* [10,30,32,74,75]. Thus, ash content varied from 26.8 ± 0.3% DW [10] to 45.2 ± 1.7% DW [30], protein content from 7.3 ± 0.3% DW [30] to 9.9 ± 0.5% DW [10], and carbohydrate content from 27.8 ± 1.0% DW [30] to 61.5 ± 0.5% DW [10]. In general terms, the characteristics of dried *S. latissima* in the study by Sappati et al. [10] are in accordance with the literature and the macroalgae seemed particularly rich in proteins, minerals, and carbohydrates. It is also interesting to note that observed differences between May and June (for ash, proteins, and carbohydrates) were not observed in the study of Sappati et al. [10]. In this work, *S. latissima* were longline cultivated in Damariscotta Bay (Maine, USA), harvested in early May and late June 2017, and dried at 50 °C for 4 h [10]. Compared to common foods, *S. latissima* provided less protein than dried lentils (25.4 g/100 g [76]), and was closer to wheat bran, which contains about 15 g/100 g of protein [77]. 

Mineral composition (Table 2) of dried seaweeds was significantly lower in Mg and Fe for crude seaweeds in May (532.00 ± 14.00 mg/100 g and 34.33 ± 2.52 mg/100 g, respectively) and higher for I in June (0.21 ± 0.01 g/100 g). No other significant differences were found in dried versus crude *S. latissima*. Between dried May and June specimens, only two differences were observed for Fe (higher in May, reflecting the mineral composition of seaweeds without treatment) and I (higher in June). Compared to blanching and steam processing, and as for ash contents, drying seaweed seems to have a favorable effect on the preservation of different minerals, probably due to the absence of leaching. Hell et al. [13] reported a Na concentration of 3.91 ± 0.09 g/100 g and a K concentration of 7.21 ± 0.22 g/100 g for dried *S. latissima*, which are close to those obtained (2.25 g/100 g on average and 11.82 g/100 g on average), and Stévant et al. [30] found a largely higher I content of 0.52 ± 0.14 g/100 g for the same macroalgae (dried at 40 °C). Another study investigated the mineral composition of two steamed and dried brown Atlantic seaweeds, *Cystoseira abies-marina* and *Zonaria tournefortii* [78]. The results indicated no differences for Na, Mg, K, Ca, and Fe for *Z. tournefortii* whereas for *C. abies-marina* the amounts of Na and K were higher for steam treatment. Those results differ from ours but highlight variations through seaweed species, environment, and applied treatment. 

### 3.2. Bioactive Compound Contents and In Vitro Bioactive Potential

#### 3.2.1. Variations through Growth Conditions of Crude *S. latissima*

- Bioactive compound content

The bioactive compound content of crude *S. latissima* cultivated on the marine farm is shown on a dry matter basis in Table 3.

No significant difference was observed in alginates, fucoidans, laminarans, and polyphenols for crude *S. latissima* blades. However, carotenoids were significantly higher in samples harvested in May compared to June, with concentrations varying between 537 and 579 μg/g, respectively. For alginate, fucoidans, and laminarans the results are in accordance with the presented fiber contents. In a previous study, seasonal variations of fucoidans in *S. longicruris* (*S. latissima*) have already been observed, not necessarily reporting the total content but mainly the proportions of constructing monosaccharides [27]. In that study, the global percentage of sulphate fucoidans was not significantly different between May, August, and November, whereas d-galactose, d-mannose, and d-galacturonic acid were significantly higher in May and d-glucose in August. Variations in the monosaccharides making up laminarans were also observed through the months, with a higher d-glucose content occurring in May and August. In the present study, since the determination of these polysaccharides was performed for the total content, seasonal variations within the monosaccharides might not be visible, but could well have occurred.

The polyphenol results were close to those obtained by Boisvert et al. [12] on *S. longricruris* (*S. latissima*), who reported a total phenolic content of 3.30 ± 0.01 mg GAE/g and did not vary as also shown by Roleda et al. [15] who found that *S. latissima* blades cultivated in spring and summer did not present any significant difference. Compared to the literature, the carotenoid content of the macroalgae in our study was 6 to 7 times higher than that reported by Boisvert et al. [12], who measured a total content of 77.57 ± 2.04 µg/g in *S. longricruris* (*S. latissima*). Carotenoids are pigments (such as fucoxantin) involved in photosynthesis [25]. Their production is therefore strongly linked to light intensity and nitrogen availability [17,55]. While the photoperiod is longer in June, the amount of nitrogen is lower [17,23,26]. These results may be related to the interaction between these factors. In addition, the rise of the longlines from 7 m to 4 m below sea level in May increases the light intensity for the cultivated macroalgae, thus inducing a higher production of carotenoids [79].

- Protein content and extraction yields of soluble *S. latissima* extracts

Extraction of water-soluble *S. latissima* extracts > 1 kDa, presented in Table 4, showed significant differences in the average extraction yields between May and June with 1.68% and 0.91% DW, corresponding to protein extraction yields (PEY) of 6.55% and 4.09%. Although the extraction yields were close to those obtained for *S. latissima* (1.0%) [13], the PEY were lower than in previous studies. Indeed Bondu et al. [54], using phosphate buffer pH 7.0, presented a PEY of 8.57% and that of Hell et al. [13] was 11.53%. Differences between the present work and the literature could be related to the processing state of macroalgae (fresh in our study versus dried in [13,54]). Moreover, a high grinding gradient can facilitate the extraction process [55] and differences could be induced by the particle size variation [80]. The objective of the extraction was to recover proteins and peptides from the macroalgae. However, this process could be limited by the interactions of these molecules with polysaccharides [81,82,83]. Between May and June, the differences could be linked to the macroalgae chemical composition, where specimens recovered in May were richer in proteins, as for the PEY.

- Antioxidant capacity of soluble S. latissima extracts: ORAC and FRAP tests

**Figure 3:**
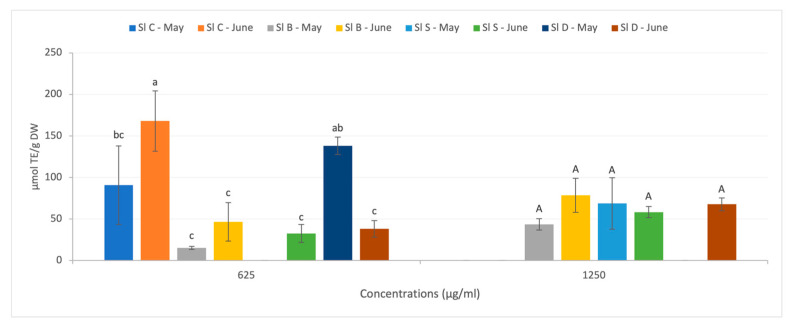
Antioxidant capacities of crude and processed *S. latissima* harvested in May and June evaluated with Oxygen Radical Absorbance Capacity (ORAC) assay. Results are presented in µmol TE/g DW at various concentrations (microgram of extract per milliliter). Each value is presented as mean ± SD (N = 3). Histogram bar means with lowercase letters (a–c) differ significantly (*p*-value < 0.05) and means with the same capital letters do not differ significantly (*p*-value > 0.05). *Sl*: *S. latissima*, C: crude, B: blanched, S: steamed, D: dried.

The ORAC assay results for water-soluble *S. latissima* extracts are presented in Figure 3. A total of eight different concentrations were tested, but only one (625 µg extract/mL) gave reliable results for crude *S. latissima* extracts. According to ANOVA statistical analysis (*p*-value < 0.0001), a significant difference was observed depending on the harvest month, with an ORAC value in May of 90.62 ± 47.16 µM TE g^−1^ DW versus 168.03 ± 36.29 µM TE g^−1^ DW in June. These results seem close to those obtained by Bondu et al. [54], where ORAC values for *S. latissima* extracts were under 250 µM TE g^−1^ DW. In another study, Stefaniak et al. [84] reported that *S. latissima* water extracts had an ORAC value of 259 ± 37 µM TE g^−1^ DW. However, our results are relatively lower than those obtained by Hell et al. [13] (328.97 ± 27.52 mM TE g^−1^ DW). 

For the FRAP assay of water-soluble *S. latissima* extracts, only one of the five selected concentrations (500 µg extract/mL) was relevant. Unlike the ORAC analysis, no significant difference (*p*-value = 0.4682) was observed between May and June (7.86 ± 1.17 and 12.21 ± 3.87 27.52 µM TE g^−1^ DW, respectively). It should be noted that the FRAP values obtained were particularly low. For instance Bondu et al. [54] obtained FRAP values between 77.53 and 131.35 µM TE g^−1^ DW for hydrolyzed protein extracts of *S. longricuris* (*S. latissima*). Differences observed in the literature could be due to different factors such as macroalgae growth conditions [14,17,18]; macroalgae state (frozen in that study versus dried in others); the extraction process which is similar to Bondu et al. [54] and Hell et al. [13] but different from Stefaniak et al. [84]; macroalgae grinding gradient [55,80] or the possible hydrolysis of extracts which can improve antioxidant capacities [85]–performed in Bondu et al. [54]. 

Water-soluble *S. latissima* extracts > 1 kDa contained peptides and proteins, but also polyphenols and polysaccharides which are also known bioactive molecules [13]. For instance, polyphenols from macroalgae have shown, among other potential bioactivities, a strong antioxidant capacity [5,14,47], as have sulfated polysaccharides (such as alginates, fucoidans, and laminarans) [7,86]. Surprisingly, our ORAC values do not vary in the same way as crude *S. latissima* or extracts. Indeed, in crude samples fibers, alginates, fucoidans, laminarans, and polyphenols were not different between May and June, and the protein extraction yield was significantly higher in May. For FRAP values, the lack of differences seems more in line with data for crude *S. latissima*, but not with the higher protein yield of May extracts. The differences in the observed results between these two antioxidant assays could be explained by the fact that the measuring mechanism is not the same [87]. In general, the antioxidant capacity of these macroalgae seems relatively low.

#### 3.2.2. Variation through Food Processing of *S. latissima*

The bioactive compound content of processed (blanched, steamed, and dried) *S. latissima* is shown in Table 3, the extraction of water-soluble extracts > 1 kDa in Table 4, and the ORAC results in Figure 3. Statistical tests showed the presence of interactions between harvest months and treatments (*p*-value < 0.05). FRAP results which did not differ significantly (*p*-value > 0.05) are not shown.

- Blanching

Some differences in potential bioactive compounds (Table 3) were observed for alginates, fucoidans, and laminarans, compared to crude seaweeds. These fibers, which are components of seaweed cell walls, can be degraded or released by heat treatment, affecting tissue integrity and texture [39,88]. However, to our knowledge, no study has been conducted on the impact of blanching on these bioactive polysaccharides. The total carotenoid content decreased by 40% between crude and blanched *S. latissima* (no significant difference between harvest months). Fucoxanthin, a major carotenoid and brown pigment known to be sensitive to heat treatment, appears to be degraded upon blanching from 60 °C (by color investigation) [33,40]. The blanching process also induced a loss of polyphenols but only in the case of macroalgae harvested in May. The literature seems more heterogeneous on this point, indeed Akomea-Frempong, Skonberg, et al. [31] observed no difference in the total polyphenol content for blanched *S. latissima*, while Nielsen et al. [41] and Akomea-Frempong, Perry et al. [39] observed a decrease with this treatment. The differences could be linked to harvest time [14,17,18] or conditions of blanching (vacuum bags vs. direct immersion, applied temperature, and duration) [31,39,41]. 

Interestingly, observed differences in extraction yield, PEY, and protein percentages of water soluble from crude *S. latissima* harvested in May and June (Table 4) disappeared with blanching. This phenomenon could be linked to the fiber content of crude seaweeds, which is similar, and the interaction between proteins and polysaccharides that could reduce protein extraction [81,83].

The ORAC test (with a concentration of water-soluble extracts of 625 µg/mL) indicated significantly higher antioxidant activity in fresh than in blanched *S. latissima* (Figure 3). No significant difference was found in blanched seaweeds between May and June (at 625 and 1250 µg/mL). For the FRAP test performed with 500 µg/mL extracts from blanched seaweeds from both harvest months (9.92 ± 8.00 µM TE g^−1^ DW in May and 7.60 ± 0.84 µM TE g^−1^ DW in June), no differences were observed between treatments or months of harvest. The FRAP results were similar to those obtained by Akomea-Frempong, Perry et al. [39], and Akomea-Frempong, Skonberg, et al. [31]. The blanching treatment does not seem to affect secondary metabolite contents that reduce Fe^3+^ into Fe^2+^. However, ORAC results could be affected by the loss of polyphenols.

- Steaming

In steamed specimens, the contents of alginates, fucoidans, and laminarans, as potential bioactive compounds (Table 3), were similar to blanched when compared to crude seaweeds. The only difference observed was for fucoidans in June, where the steamed seaweed amount (9.50 ± 0.61 g/100 g) was significantly higher than blanched (7.30 ± 0.44 g/100 g). The higher proportions of alginates and fucoidans in steamed seaweed than in crudes could be linked to cellular disruption through heat [89]. Interestingly, the carotenoid content was significantly the lowest in June (337.00 ± 17.00 μg/g) and the polyphenol content the lowest in May (2.85 + 0.07 mg GA/g) compared to fresh seaweeds, thus dependent on the harvest month. Previous work that performed hydrothermal processing (autoclave–100 °C, 15 min) on *Laminaria saccharina* (another name for *S. latissima*) reported a total phenolic content that was higher in fresh macroalgae [89]. Another study conducted by Susanto et al. [33] on steamed (100 °C–15 min) *Sargassum iliciform* showed no significant difference between fresh and steamed seaweed, but the amount seemed lower for steamed. Carotenoids (fucoxanthin and β-carotene) of seaweeds have been investigated in the literature. Indeed, Amorim-Carrilho et al. [44] detected a lower amount of fuconxanthin in steamed (40 min) versus fresh *Himanthlia elongata* seaweeds and no difference in β-carotene. The study also investigated carotenoids in boiled *H. elongata* (100 °C–15 min) and showed higher fucoxanthin and β-carotene contents in blanched seaweeds. These results differ from ours, where the carotenoid content is better preserved in steamed *S. latissima*. The difference could be due to seaweed species, growth environment [5,14,89], or applied treatments and heat [44]. 

As with blanching, the initial differences observed for PEY in crude macroalgae were eliminated by steaming (Table 4). PEY were not different between steaming and blanching, and neither between May and June for steamed specimens. 

No significant difference between treatment and harvest month was observed for antioxidant potential, as indicated by ORAC (1250 µg/mL–Figure 3) and FRAP tests (500 µg/mL, 11.91 ± 2.94 µM TE g^−1^ DW in May and 8.69 ± 1.23 µM TE g^−1^ DW in June). However, at 650 µg/mL, the ORAC results for steamed June specimens were significantly lower than those for crude seaweeds. To date, little information is available on the antioxidant potential of steamed seaweed. However, Rajauria et al. [89] found a higher FRAP activity in steamed (100 °C, 15 min) *Laminaria saccharina* (*S. latissima*), *Himenthalia elongata*, and *Laminaria digitata* than in fresh macroalgae.

- Drying

The potential bioactive compound content for dried seaweeds (Table 3) was not significantly different from crude seaweeds for sulfated polysaccharides (alginates, fucoidans, laminarans). The results are similar to those obtained for dried *S. latissima* by Stévant et al. [30] for alginates and laminarans (about 14 g/100 g and 1.5 g/100 g, respectively) and by Albers et al. [32] for alginates (18.9 ± 0.1 g/100 g), but not for laminarans since these authors found the content to be higher (13.2 ± 0.2 g/100 g). On the other hand, carotenoid (405.00 ± 11.00 μg/g in May and 330.00 ± 28.00 in June) and polyphenol contents (2.60 ± 0.20 mg GAE/g in May and 2.33 ± 0.32 in June) were lower than in crude seaweeds. Carotenoids and polyphenols are highly sensitive to heat and could be negatively affected by the drying treatment [10,34]. In previous work performed on *Fucus vesiculosus*, *Fucus distichus* and *Ascophyllum nodosum*, three brown seaweeds, drying treatment (20 °C for 5 days) resulted in a lower total phenolic content compared to fresh seaweed and the effect on fucoxanthin (carotenoid) was variable with different species [35]. In another study, compared to the fresh state, the brown macroalgae *Phyllaria reniformis* had higher total carotenoid and fucoxanthin contents with drying treatment (38 °C for one week) [90]. The effect of drying seems clearly dependent on the seaweed species and the temperature/duration of treatment. It was also observed that carotenoids varied according to the month of harvest, with a higher content in dried *S. latissima* harvested in May (405.00 ± 11.00 μg/g), but the same was not true for polyphenols, which were not significantly different between May and June. This finding differs from previous literature observations. Indeed, Sappati et al. [10] reported a significantly higher amount of total phenolic content for *S. latissima* harvested in early May versus late June. Compared to other heat treatments (blanching and steaming) the effect of the drying process seems highly dependent on the harvest month, with the values being close in May, and lower for dried seaweeds in June. In general, the impact of temperature on carotenoids and polyphenols (100 °C for blanching and steaming versus 40 °C for drying) seems similar and deleterious. 

Surprisingly, for water-soluble dried *S. latissima* extracts (Table 4), even if protein percentages were not different from crude extracts, the PEY was lower in May (3.78 ± 0.32%) and higher in June (6.82 ± 0.48%). Polysaccharides are known to limit protein extraction [81,83], but in the case of dried *S. latissima*, the fiber content between May and June was not significantly different and only carbohydrates were slightly, but significantly higher in June.

For antioxidant activity, no significant differences were observed for ORAC tests (1250 µg/mL–Figure 3) or FRAP tests (9.28 ± 2.04 µM TE g^−1^ DW in May and 9.47 ± 4.78 µM TE g^−1^ DW in June for a concentration of 500 µg/mL) with crudes versus dried and May vs. June. However, at a concentration of 625 µg/mL extract, the ORAC results were significantly higher in dried *S. latissima* harvested in May compared to June, and similar to crude extracts. This phenomenon could be related to the higher carotenoid content in May.

## 4. Conclusions

Longline cultivated *S. latissima* cultivated on a marine farm in Baie-des-Chaleurs (Quebec) showed a strong seasonality with growth markers during the May period and with reproductive markers, or at least a transition between these two phases, in June. Indeed, May specimens were significantly richer in proteins (11.00 ± 0.14%), ash (43.06 ± 0.07%), iodine (0.20 g/100 g), iron (41.67 ± 2.89 mg/100 g), and carotenoids (558.00 ± 21.00 μg/g) which are related to active photosynthesis and growth, whereas the carbohydrate content (47.82 ± 0.52%) was significantly higher in June. No significant difference was found in the fiber content for both harvested months, thus the rise of carbohydrates in June should be related to a greater synthesis of small sugars. Antioxidant potential was higher with the ORAC test (water-soluble extract concentration of 625 µg/mL) in samples harvested in June but no significant difference was found with the FRAP test (water-soluble extract concentration of 500 µg/mL). Variations in chemical and mineral composition, potential bioactive compounds of macroalgae samples, and potential antioxidant activity of seaweed extracts were thus strongly influenced by environmental factors such as nutrient availability, temperature, light exposure, etc. In order to produce the best food quality for customers, harvesting *S. latissima* in May might be the best option. On the other hand, measurements of macroalgae growth and yield by both months of harvest, which were not explored in this study, would complement the data presented here. For processed *S. latissima*, interactions between the harvest month and processing were clearly detected (*p*-value < 0.05), showing the different impacts of blanching, steaming, and drying according to the month of harvest. The chemical and mineral composition was better preserved with the drying process, with the highest ash content (42.96 ± 0.15%) and antioxidant potential occurring in May. However, the deleterious impact of heat on carotenoids and polyphenols was observed even with a low-temperature drying process. Both blanching and steaming processes resulted in massive *S. latissima* mineral content leaching (loss of about 50% of ash content). Thus, the protein and carbohydrate contents were significantly higher, but since the results were expressed in proportions on a dry weight basis, this looks like a rebalancing of proportions. It should be noted, however, that in view of mineral composition, blanching causes higher leaching of iodine, potassium, calcium, magnesium, and iron than steaming. The deleterious impact of heat on carotenoids and polyphenols was also observed for those methods, with the highest losses detected on blanched samples. The proportions of alginates and fucoidans were significantly higher in both months in blanched and steamed samples. FRAP tests (water-soluble extract concentration of 500 µg/mL) and ORAC analysis (water-soluble extract concentration of 625 µg/mL and 1250 µg/mL) did not show significant differences between blanched and steamed processed samples. However, ORAC analysis (625 µg extracts/mL) for May and June showed losses in antioxidant potential compared to control samples. Overall, based on the results from this study, to maintain *S. latissima* composition and produce the best food quality for customers, using a drying process for seaweeds harvested in May could be the best option. However, the impact of the drying process followed by rehydration on *S. latissima* compositions should be investigated, and since the control condition for this study was frozen, comparing results with raw specimens could provide new information to better understand the impact of processing. Moreover, the next step will be to evaluate the impact of seaweed harvest month and processing method through a sensory evaluation by panelists, which will help in the final choice of seaweed harvest and processing conditions. 

## Figures and Tables

**Table 1:** Chemical composition of crudes and processed blades from cultivated *S. latissima* harvested in May 2019 and June 2019.

*Sl* Treatment and Harvest Time	Lipids (%)	Proteins (%)	Ash (%)	Carbohydrates (%)	Fibers (%)
Crudes–May	1.88 ± 0.02 ^ab^	11.00 ± 0.14 ^d^	43.06 ± 0.07 ^a^	44.05 ± 0.29 ^e^	34.57 ± 0.18 ^a^
Blanched–May	1.59 ± 0.22 ^ab^	15.46 ± 0.21 ^a^	21.67 ± 0.01 ^f^	61.28 ± 0.26 ^b^	54.77 ± 1.13 ^a^
Steamed–May	1.40 ± 0.19 ^b^	14.66 ± 0.25 ^b^	21.88 ± 0.18 ^f^	62.06 ± 0.27 ^b^	52.24 ± 0.50 ^a^
Dried–May	1.80 ± 0.24 ^ab^	10.38 ± 0.39 ^d^	42.96 ± 0.15 ^a^	44.86 ± 0.44 ^e^	34.78 ± 0.30 ^a^
Crudes–June	1.63 ± 0.16 ^ab^	9.00 ± 0.32 ^e^	41.55 ± 0.01 ^b^	47.82 ± 0.52 ^d^	37.72 ± 0.36 ^a^
Blanched–June	0.69 ± 0.04 ^c^	13.16 ± 0.47 ^c^	27.53 ± 0.03 ^d^	58.62 ± 0.67 ^c^	53.50 ± 1.17 ^a^
Steamed–June	1.30 ± 0.26 ^b^	13.13 ± 0.11 ^c^	22.24 ± 0.17 ^e^	63.34 ± 0.09 ^a^	57.12 ± 0.90 ^a^
Dried-June	1.98 ± 0.22 ^a^	9.42 ± 0.11 ^e^	40.09 ± 0.11 ^c^	48.51 ± 0.42 ^d^	35.94 ± 0.32 ^a^

Lipids, proteins, ash, carbohydrates, fibers are expressed as a percentage of the dry weight (DW) of macroalgae (mean ± SD; N = 3). Means within each column with different letters (a–f) differ significantly (*p* < 0.05). *Sl*: *S. latissima*.

**Table 2:** Mineral composition of crudes and processed blades from cultivated *S. latissima* harvested in May 2019 and June 2019.

Sl Treatment and Harvest Time	I (g/100 g)	K (g/100 g)	Na (g/100 g)	Ca (mg/100 g)	Mg (mg/100 g)	Fe (mg/100 g)
Crudes–May	0.20 ± 0.00 ^ab^	15.74 ± 1.28 ^a^	2.33 ± 0.06 ^c^	614.00 ± 22.00 ^ab^	623.00 ± 23.00 ^bc^	41.67 ± 2.89 ^a^
Blanched–May	0.01 ± 0.00 ^e^	0.95 ± 0.01 ^c^	6.20 ± 0.20 ^b^	744.00 ± 6.00 ^ab^	207.00 ± 4.00 ^f^	19.33 ± 1.53 ^c^
Steamed–May	0.06 ± 0.00 ^d^	5.51 ± 0.09 ^abc^	1.03 ± 0.12 ^d^	976.00 ± 79.00 ^a^	634.00 ± 46.00 ^bc^	31.67 ± 1.53 ^b^
Dried–May	0.19 ± 0.00 ^b^	12.70 ± 0.43 ^abc^	2.23 ± 0.06 ^c^	543.00 ± 15.00 ^bc^	532.00 ± 14.00 ^e^	34.33 ± 2.52 ^b^
Crudes–June	0.17 ± 0.01 ^c^	11.15 ± 0.13 ^abc^	2.57 ± 0.06 ^c^	638.00 ± 16.00 ^ab^	608.00 ± 2.00 ^cd^	12.33 ± 0.58 ^e^
Blanched–June	0.02 ± 0.00 ^e^	1.77 ± 0.4 ^bc^	6.97 ± 0.38 ^a^	626.00 ± 19.00 ^ab^	181.00 ± 7.00 ^g^	12.00 ± 0.00 ^e^
Steamed–June	0.07 ± 0.00 ^d^	5.20 ± 0.09 ^abc^	0.97 ± 0.06 ^d^	922.00 ± 4.00 ^a^	505.00 ± 27.00 ^e^	13.67 ± 0.58 ^de^
Dried-June	0.21 ± 0.01 ^a^	10.95 ± 0.21 ^abc^	2.27 ± 0.29 ^d^	641.00 ± 9.00 ^ab^	547.00 ± 15.00 ^de^	15.33 ± 0.58 ^d^

Iodine (I), potassium (K), sodium (Na) are expressed as g/100 g of the DW of macroalgae and calcium (Ca), magnesium (Mg) and iron (Fe) as mg/100 g (mean ± SD; N = 3). Means within each column with different letters (a–g) differ significantly (*p* < 0.05). *Sl*: *S. latissima*.

**Table 3:** Potential bioactive compound contents of crude and processed cultivated *S. latissima* harvested in May and June 2019.

Sl Treatment and Harvest Time	Alginates (g/100 g)	Fucoidans (g/100 g)	Laminarans (g/100 g)	Carotenoids (μg/g)	Polyphenols (mg GA/g)
Crudes–May	12.53 ± 2.07 b	5.47 ± 0.49 de	3.60 ± 0.44 bc	558.00 ± 21.00 a	3.77 ± 0.06 ab
Blanched–May	30.73 ± 1.50 a	8.13 ± 0.76 ab	3.73 ± 0.29 bc	323.00 ± 1.00 de	1.73 ± 0.12 e
Steamed–May	27.77 ± 1.92 a	8.13 ± 0.80 ab	2.80 ± 0.53 cd	555.00 ± 24.00 a	2.85 + 0.07 cd
Dried–May	12.57 ± 2.11 b	6.07 ± 0.75 cd	4.47 ± 0.21 ab	405.00 ± 11.00 c	2.60 ± 0.20 cd
Crudes–June	15.97 ± 1.41 b	3.83 ± 0.25 e	4.00 ± 0.10 b	466.00 ± 14.00 b	3.80 ± 0.20 ab
Blanched–June	28.30 ± 1.74 a	7.30 ± 0.44 bc	1.87 ± 0.67 de	281.00 ± 16.00 e	3.20 ± 0.35 bc
Steamed–June	28.17 ± 0.12 a	9.50 ± 0.61 a	1.07 ± 0.15 e	337.00 ± 17.00 d	4.33 ± 0.32 a
Dried-June	15.47 ± 2.14 b	4.30 ± 0.40 e	3.90 ± 2.27 b	330.00 ± 28.00 de	2.33 ± 0.32 de

Alginates, fucoidans, and laminarans are expressed as g/100 g of the dry weight (DW) of macroalgae, carotenoids as μg/g, and polyphenols as mg GA/g (mean ± SD; N = 3). Means within each column with different letters (a–e) differ significantly (*p* < 0.05). *Sl*: *S. latissima*.

**Table 4:** Protein content, extraction and protein yield of water-soluble extracts of crude and processed cultivated *S. latissima* harvested in May and June 2019.

*Sl* Extracts	Crudes–May	Blanched–May	Steamed–May	Dried–May	Crudes–June	Blanched–June	Steamed–June	Dried–June
Proteins (%)	40.68 ± 1.43 ^ab^	32.76 ± 1.56 ^d^	37.57 ± 0.89 ^bc^	42.91 ± 0.15 ^a^	36.87 ± 1.20 ^c^	35.78 ± 1.63 ^cd^	38.50 ± 1.04 ^bc^	38.27 ± 1.57 ^bc^
Extraction yield (%)	1.68 ± 0.28 ^a^	1.33 ± 0.16 ^ab^	1.40 ± 0.24 ^ab^	0.84 ± 0.10 ^bc^	0.91 ± 0.18 ^bd^	1.51 ± 0.22 ^a^	0.69 ± 0.24 ^c^	1.59 ± 0.06 ^a^
Protein extraction yield (%)	6.55 ± 1.11 ^a^	2.98 ± 0.23 ^bc^	3.76 ± 0.69 ^bc^	3.78 ± 0.32 ^bc^	4.09 ± 0.95 ^bc^	4.40 ± 0.73 ^b^	2.19 ± 0.71 ^c^	6.82 ± 0.48 ^a^

Proteins, extraction yield and protein yield are expressed as a percentage of the dry weight (DW) of macroalgae (mean ± SD; N = 3). Means within each row with different letters (a–d) differ significantly (*p* < 0.05). *Sl*: *S. latissima*.

## Data Availability

The datasets analyzed in this study are available in a publicly accessible repository that can be found here: Merinov, & Université du Québec à Rimouski. (2022). Banque des données sur les macroalgues marines des sites de la Gaspésie (2019) [Data set]. https://catalogue.ogsl.ca/dataset/ca-cioos_cbf7730a-7074-4a37-92c8-0e549d6c6194?local=fr, accessed on 6 April 2023.
